# L’érysipèle sur cicatrice post opératoire en traumatologie: à propos d'un cas et revue de la littérature

**DOI:** 10.11604/pamj.2015.21.10.6017

**Published:** 2015-05-05

**Authors:** Hatim Abid, Mohamed El Idrissi, Mohamed Shimi, Abdelhalim El Ibrahimi, Abdelmajid El Mrini, Nissrine Amraoui, Fatima Zohra Mernissi

**Affiliations:** 1 Service de Chirurgie Ostéo-Articulaire B4, CHU Hassan II, Fès, Maroc; 2 Service de Dermatologie-Vénérologie CHU Hassan II, Fès, Maroc

**Keywords:** Erysipèle post opératoire, cicatrice, jeune, traumatologie, postoperative Erysipelas, scar, young, traumatology

## Abstract

L’érysipèle sur cicatrice post opératoire est une entité rare, décrite principalement chez des patients présentant les facteurs de risque classiques de la maladie au niveau des sites de veinectomie pour pontage coronarien. En traumatologie orthopédie, nous n'avons que les 3 cas rapportés dans le travail de Dhrif survenus au décours d'une implantation prothétique chez des malades à risque. Nous présentons à travers cet article, le cas d'un érysipèle post opératoire sur une cicatrice d'ostéosynthèse d'une fracture fermée du pilon tibial, ayant la particularité du terrain et des circonstances de survenues, pour enfin conclure aux principes de la prévention primaire à adopter.

## Introduction

L’érysipèle est une dermo-hypodermite aigue non nécrosante d'origine le plus souvent streptococcique. Il touche dans plus de 85% des cas les membres inférieurs en étant favorisé par la présence d'une porte d'entrée le plus souvent cutanée. D'un point de vue épidémiologique, on considère l'existence d'une porte d'entrée, le lymphoedème et l'obésité comme les principaux facteurs de risque de survenue [1]. Dans la littérature, cette entité pathologique a été rarement décrite sur une cicatrice cutanée post opératoire en dehors de quelques cas apparus sur des sites de veinectomie pour des pontages coronariens [2, 3]. Nous rapportons dans ce travail le cas d'un jeune patient ayant présenté un érysipèle de la jambe sur une cicatrice opératoire d'une ostéosynthèse pour une fracture fermée du pilon tibial, dans le but d’étudier les particularités épidémiologiques, cliniques, thérapeutiques et étiopathogéniques de cette entité chez des patients de traumatologie.

## Patient et observation

**Figure 1:**
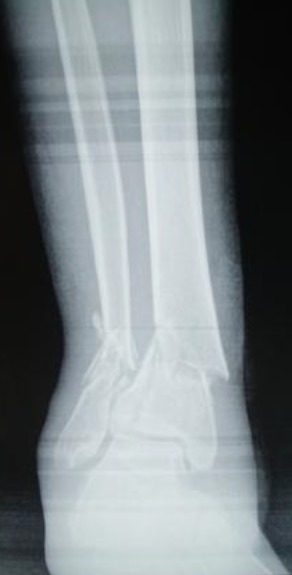
Radiographie de la jambe droite de face montrant la fracture du pilon tibial

**Figure 2:**
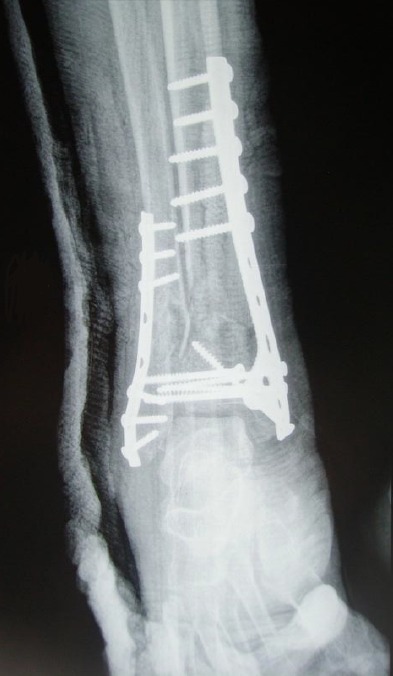
Radiographie de contrôle après à 2 mois du post opératoire n'objectivant pas de signes de sepsis sur matériel

Il s´agit d'un patient âgé de 28 ans, ayant bénéficié d'une ostéosynthèse d'une fracture fermée du pilon tibial par plaque vissée (Figure 1, Figure 2) il y a 2 mois avec des suites post opératoires simples, sans épisode antérieur d’érysipèle, qui a présenté de façon brutale une grosse jambe droite rouge et douloureuse évoluant dans un contexte de fièvre, de frissons et de conservation de l´état général. L'examen général trouvait un patient fébrile à 38,5° avec sur le plan cutané, un placard érythémateux chaud et douloureux, œdémateux au niveau des 2/3 inférieurs de la jambe droite, s´étendant jusqu´au pied, bien limité prenant la cicatrice opératoire avec quelques lésions purpuriques et nécrotiques, sans crépitations ni troubles de la sensibilité ni fistulisation, sans lymphoedème ni intertrigo inter-orteil. Le reste de l'examen somatique avait objectivé une adénopathie inguinale homolatérale sensible de 0.8 cm. Sur le plan biologique, le bilan avait montré une hyperleucocytose à polynucléaires neutrophiles à 18800/mm3 et une augmentation de la protéine C réactive à 322 mg/l ceci avec une glycémie à jeun de valeur normale. L'exploration radiologique de la jambe a mis en évidence une fracture en voie de consolidation sans signes radiologiques de sepsis sur le matériel d'ostéosynthèse. Dès lors le diagnostic retenu était celui d'un érysipèle post opératoire. Le patient a été mis sous amoxicilline protégée par voie injectable avec repos et surélévation du membre atteint. Une amélioration clinique et biologique a été notée, d'où le passage à la voie orale au troisième jour du traitement. Le patient a bénéficié de 21 jours d'antibiothérapie. L’évolution a été marquée par la disparition complète du placard et de l’œdème avec un recul de 8 mois sans récidive. Après consolidation, le patient a bénéficié d'une ablation du matériel d'ostéosynthèse.

## Discussion

L’érysipèle sur cicatrice chirurgicale a été rarement rapporté dans la littérature. En effet, rares sont les cas qui ont été publiés depuis les premières descriptions de cette entité pathologique par Baddour et al en 1982 [2]. A ce propos, cet auteur avait traité en 1997 une série de 5 malades cardiaques, ayant développé un érysipèle des membres inférieurs sur des cicatrices de veinectomie pour pontage coronarien [4]. Dans les mêmes circonstances de comorbidité (malades cardiaques), mais pour une série plus importante, Karakas [5] de sa part avait publié en 2002 un travail qui a porté sur 31 cas. En chirurgie traumatologique et orthopédique, nous n'avons pu trouver dans les archives de la littérature que les résultats du travail de Dhrif [6] ayant colligé, sur son étude rétrospective réalisée entre 1999 et 2003, 3 cas survenus dans les suites d´une implantation de prothèse ostéoarticulaire chez des patients d'un âge moyen de 61ans, et dont un était porteur d'une insuffisance veineuse chronique. De notre part, le cas du patient que nous rapportons a la particularité de l’âge jeune qui est de 28 ans, chez qui l’érysipèle s'est installé au décours d'une ostéosynthèse par plaque vissée pour une fracture fermée du pilon tibial. Sur le plan physiopathologique, la plupart des auteurs [1, 7] considèrent la cicatrice opératoire comme source d'altération du drainage lymphatique et par conséquent un facteur de risque de la survenue de l’érysipèle. Chez notre malade, le choix de la voie d'abord était conditionné par le type de la fracture qui nous a imposé une approche antéro interne du tiers inférieur de la jambe, dont l'irrigation et le drainage sont précaires pour des raisons anatomiques. L'incidence est d'autant plus importante en présence des autres facteurs classiques à savoir le diabète, l'obésité et le lymphoedème qui manquaient chez notre malade, chez qui l'apparition de l’érysipèle semble être due à l'association concomitante d'une porte d'entrée cutanée et d'un défaut du drainage au niveau de la microcirculation lymphatique locale, détruite en per-opératoire lors de l'accès chirurgical de cette zone fragile. Dans la littérature, l'apparition de l’érysipèle peut se faire aussi bien de façon précoce que tardive par rapport à la date de la cicatrice. Le délai oscille entre 2 mois à 2 ans dans la série de Baddour [4], et varie dans celle de Hattab [8] entre 8 mois à 26 ans. Ces auteurs rattachent la précocité de l'installation de l’érysipèle chez des malades par rapport à d'autres, à la coexistence de facteurs de risques multiples et à la comorbidité des malades âgés. Dans notre contexte, le placard érythémateux était survenu 2 mois après la chirurgie, ce qui est un délai précoce que nous expliquons en l'absence des facteurs de risques classiques, par un défaut de drainage lymphatique, initié par l'approche à ciel ouvert de la fracture, puis aggravé par le non appui qui est recommandé pendant 8 à10 semaines en cas de fractures du pilon tibial traitée par plaque vissée.

**Figure 3:**
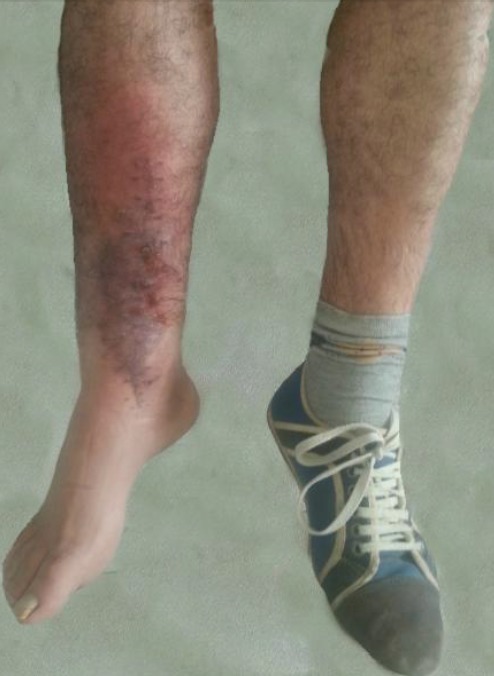
La présentation clinique de l’érysipèle sur cicatrice opératoire

Cliniquement, l’érysipèle sur cicatrice opératoire présente les mêmes caractéristiques que l’érysipèle sur peau saine [1, 4, 5]. Il s'agit d'un placard inflammatoire érythémateux douloureux chaud englobant la cicatrice cutanée. Les adénopathies satellites sont présentes dans près de 30% des cas et le lymphoedème préexistant est rapporté avec une incidence de 20% [1, 9]. Chez notre malade, la présentation clinique était typique faite de l'association d'un placard d’érythème (Figure 3) et d'une adénopathie inguinale homolatérale sensible. Dans la littérature, le traitement antibiotique de l’érysipèle sur cicatrice opératoire ne nécessitera ni une durée plus prolongée, ni des doses plus importantes de pénicilline. La durée moyenne de l'antibiothérapie est de 15 jours dans les formes simples. Elle est plus prolongée atteignant les 21 jours chez les sujets avec des comorbidités sous jacentes [1, 7, 9]. Pour notre malade, jeune et sans antécédents pathologiques notables, nous avons opté pour une antibiothérapie prolongée du fait du risque potentiel d'un sepsis sur matériel d'ostéosynthèse dont la survenue nous aurait imposé à ce stade de non consolidation, l'ablation de la plaque et la mise en place d'un fixateur externe. D'une façon générale, l’érysipèle des membres inférieurs expose à des risques d'ordre hématologique représentés par la maladie thromboembolique, et d'autres de nature infectieuse à l'exemple de l'abcès et plus rarement de la faciite nécrosante. A distance du premier épisode, la plupart des auteurs soulèvent la problématique majeure du lymphoedeme résiduel et de la récidive qui est observée dans 6 à 57% des cas selon les séries et qui survient chez des malades à risque [7, 10]. Dans la littérature, la récidive se déclare chez 12% des patients à 6 mois, 28.5% à 2 ans et 30% des cas à 3 ans dans un contexte d'intertrigo inter orteil persistant et surtout d'une cicatrice opératoire infectée [10]. Chez notre malade, nous ne déplorons aucunes complications jusqu´à 8 mois de suivi du fait de la mise systématique de nos opérés des membres inférieurs sous anti coagulation préventive tout au long de la période de la décharge et de l'antibiothérapie que nous avons maintenue de façon prolongée en raison de la présence du matériel d'ostéosynthèse. En terme de prévention, les travaux qui se sont intéressés à ce sujet insistent sur l'intérêt majeur de la prise en charge adaptée de la stase veineuse et lymphatique assez fréquente chez les opérés des membres inferieurs, d'abord par la kinésithérapie de drainage lymphatique manuel qu'on peut démarrer dans les suites post opératoires immédiates, puis par le port de bas de contention de façon alternée [11, 12]. Dans le cas que nous rapportons, ces mesures préventives étaient prescrites à la sortie sans pour autant qu'elles soient respectées par le malade.

## Conclusion

L’érysipèle sur cicatrice post opératoire est une complication rarement décrite en chirurgie traumatologique. Elle présente les mêmes caractéristiques cliniques et thérapeutiques que l’érysipèle sur peau saine. Sa gravité est majorée par la présence du matériel d'ostéosynthèse vue le risque de sepsis. De ce fait, la prévention primaire de cette entité pathologique, qui passe par des mesures simples, doit faire l'objet d'une prescription systématique.
